# Colony-live *—*a high-throughput method for measuring microbial colony growth kinetics*—* reveals diverse growth effects of gene knockouts in *Escherichia coli*

**DOI:** 10.1186/1471-2180-14-171

**Published:** 2014-06-26

**Authors:** Rikiya Takeuchi, Takeyuki Tamura, Toru Nakayashiki, Yuichirou Tanaka, Ai Muto, Barry L Wanner, Hirotada Mori

**Affiliations:** 1 Graduate School of Biological Sciences, Nara Institute of Science and Technology, Ikoma, Nara, Japan; 2 Bioinformatics Center, Institute for Chemical Research, Kyoto University, Uji, Kyoto, Japan; 3 Graduate School of Agricultural Science, Kobe University, Nada, Kobe, Japan; 4 Department of Biological Sciences, Purdue University, West Lafayette, Indiana 47907, USA

**Keywords:** Growth kinetics, Phenotype screening, High-throughput, Single-gene knockout, Keio collection, Lag time of growth (LTG), Maximum growth rate (MGR), Saturation point growth (SPG)

## Abstract

**Background:**

Precise quantitative growth measurements and detection of small growth changes in high-throughput manner is essential for fundamental studies of bacterial cell. However, an inherent tradeoff for measurement quality in high-throughput methods sacrifices some measurement quality. A key challenge has been how to enhance measurement quality without sacrificing throughput.

**Results:**

We developed a new high-throughput measurement system, termed Colony-live. Here we show that Colony-live provides accurate measurement of three growth values (lag time of growth (LTG), maximum growth rate (MGR), and saturation point growth (SPG)) by visualizing colony growth over time. By using a new normalization method for colony growth, Colony-live gives more precise and accurate growth values than the conventional method. We demonstrated the utility of Colony-live by measuring growth values for the entire Keio collection of *Escherichia coli* single-gene knockout mutants. By using Colony-live, we were able to identify subtle growth defects of single-gene knockout mutants that were undetectable by the conventional method quantified by fixed time-point camera imaging. Further, Colony-live can reveal genes that influence the length of the lag-phase and the saturation point of growth.

**Conclusions:**

Measurement quality is critical to achieving the resolution required to identify unique phenotypes among a diverse range of phenotypes. Sharing high-quality genome-wide datasets should benefit many researchers who are interested in specific gene functions or the architecture of cellular systems. Our Colony-live system provides a new powerful tool to accelerate accumulation of knowledge of microbial growth phenotypes.

## Background

High-throughput growth measurements with single-gene knockout (SKO) collections have enabled genome-wide studies to illuminate cellular systems, such as comprehensive genetic interactions [1-4] and chemical-genetic interactions [5]. However, an inherent tradeoff between throughput and measurement quality means that high-throughput methods sacrifice some measurement quality. Measurement quality can be assessed by small variations in different experiments, reproducibility independent of plate differences or colony position on the plate, and the amount of information generated. These are critical to achieving the resolution required to identify unique phenotypes among a diverse range of phenotypes. A key challenge is how to enhance measurement quality without sacrificing throughput.

We considered two critical problems of a current high-throughput growth measurement method. The first problem is the neighbor effect. To date arrayed-colonies have been used for high-throughput growth quantification because experiment throughput is dramatically increased by manipulating high-density arrayed colonies (1536 colonies/plate) using robotic technology [6]. Since this method can provide cost-effective growth measurement, high-density arrays have been widely used in phenotype screening studies of genome-wide mutant library [1-5]. Despite the throughput advantage, growth inhibition by neighboring colonies (the neighbor effect) arises due to insufficient separation between the arrayed colonies. Although the neighbor effect is likely associated with two general factors: competition for nutrients and cell-cell communication (quorum sensing), the specific cause is usually unknown. The magnitude of the neighbor effect varies widely for colony position and between plates. Thus, the neighbor effect interferes with growth quantification. A normalization of systematic biases generated by the neighbor effect has been successful for improved measurement accuracy and precision [7]. However, this normalization procedure was designed for the conventional colony quantification method. We therefore developed a new normalization method that is suitable for high-throughput systems which differs markedly from the conventional method [7].

The second problem is the lack of information about growth kinetics. The conventional method measures colony area at a specified time point [8], so that growth kinetics of the colony is completely overlooked. Growth kinetics provides rich information, and enables detailed classification by addressing three distinct growth characteristics: lag time, growth rate, and growth yield [9]. Such classification can be used to elucidate functions active at certain growth phases, natural trait variation [10,11], rare phenotypic variants [12], and growth strategies [13]. While several methods are currently available for measuring growth kinetics of colonies [12-15], these methods are generally not useful in high-throughput measurement because they do not consider the neighbor effect described above.

Here, we developed a high-throughput measurement system, termed Colony-live, which provides growth kinetics information with a new normalization method against the neighbor effect. To demonstrate the utility of the Colony-live system, we measured growth kinetics of the entire Keio collection of *Escherichia coli* K-12 SKO mutants [16,17]. Colony-live successfully detected slow-growth phenotypes of mutants that were undetectable by the conventional method. In addition, growth kinetics information elucidated genes that are physiologically important in the early growth phase and for efficient growth. Taken together, our new method provides additional insights into reliable physiological study at a genome-wide level.

## Results

### Development of the colony-live system

**Figure 1:**
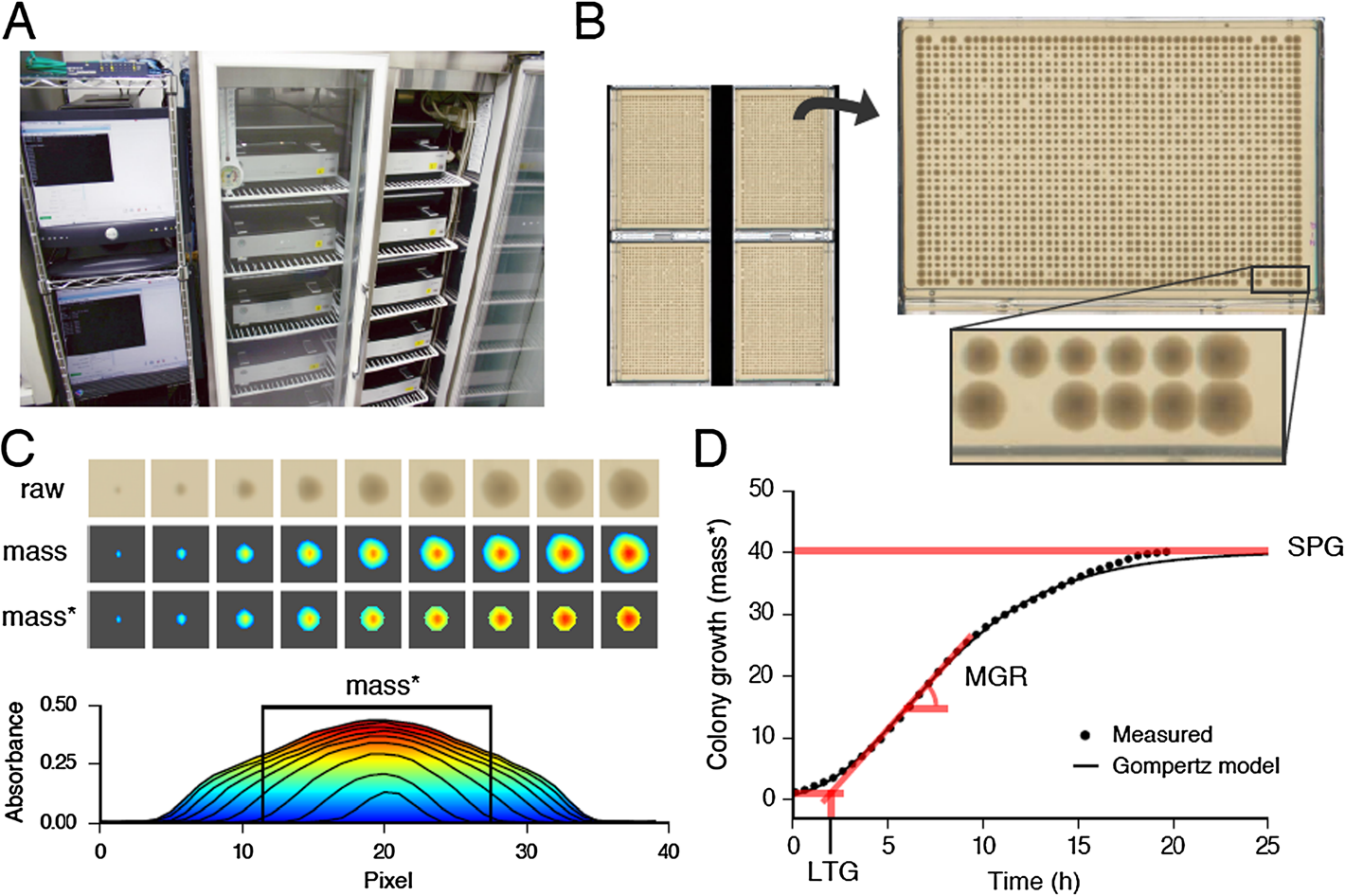
**An overview of the Colony-live system. (A)** Scanners in incubator. **(B)** Each scanned image contains four plates in which 1536 colonies were grown. **(C)** Example of image analysis. (Upper) Raw and analyzed images of a growing colony at 9 different incubation time points (2.5-h intervals; from 0 h to 20 h). Colony growth was quantified by integrating the mass of the whole (mass) or the center (mass*) region of a colony. Colors represent pixel absorbance values. (Lower) A cross section view of a growing colony at 9 time points corresponding to the upper images. The mass* region is indicated. **(D)** Example of growth analysis. Time-dependent mass* values (dots) of a growing colony were regressed to the Gompertz model (black line) and summarized into lag time of growth (LTG), maximum growth rate (MGR), and saturation point of growth (SPG). This example is an *ackA* mutant showing longer LTG for better display of LTG.

We developed the Colony-live system by combining commercially available devices and in-house software. We confirmed the sufficient quantitative performance of a commercial scanner based on the comparison to OD_600_ values measured by Microplate Reader (see Additional file 1: Figure S1). For parallel growth measurement, we installed 12 scanners in an incubator (Figure 1A). Four standard size rectangle plates can be scanned by each scanner (Figure 1B). Thus, the maximum capacity is 48 plates, which can provide colony growth curves for more than seventy thousand colonies simultaneously. To monitor colony growth kinetics, colony growth was captured by periodic scans every 30 min over a 20 h incubation period (see Additional file 2: Movie). Scanning was performed with transmitted light to enable quantification of colony mass in the center region (Figure 1C, mass*), which we show below provided a novel quantification metric. Time series data were fitted to the Gompertz population growth model [9], and three growth characteristic value were calculated: LTG (lag time of growth), MGR (maximum growth rate), and SPG (saturation point of growth; Figure 1D). All data including experimental information, measurement time, and analytical values were stored in MySQL database for secure and efficient data management.

### Normalization to reduce colony neighbor effects

**Figure 2:**
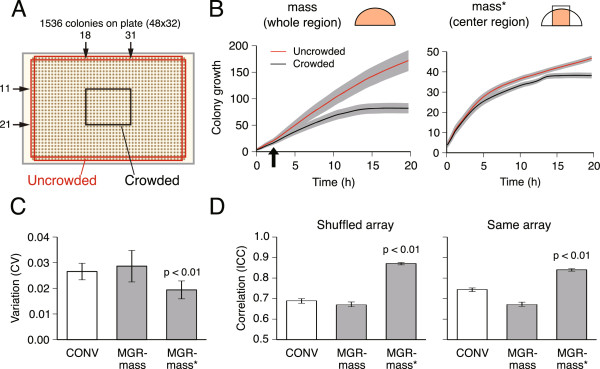
**Improvement of measurement by reducing the neighbor effect. (A)** Position of crowded (row = 11-21 × column = 18-31) and uncrowded (row = 1, 32 × column = 1, 48 without corner) colonies. **(B)** Growth of colonies in two positions was monitored to assess the neighbor effect. The average (line) and SD (gray area) of wild-type colony growth in the two positions are shown. Colony growth was quantified by integrating the mass of the whole (mass) or the center (mass*) region of each colony. **(C)** Growth values were determined by the conventional method (CONV; white bar) and Colony-live (MGR; gray bars), and the MGR values were calculated based on the mass (MGR-mass) and mass* (MGR-mass*) values. The coefficient of variation (CV) of growth values for 1536 wild-type colonies within a plate was determined using 5 independently prepared plates. **(D)** Intra-class correlation (ICC) of multiple growth measurements for the Keio mutant collection was evaluated in two experimental designs: the colony array format of the Keio collection was the same (Same array, 7 independent experiments for entire set of Keio collection) or randomly shuffled (Shuffle array, randomly 4 plates) for each measurement. Error bars represent 95% confidence intervals.

Emergence of the neighbor effect was confirmed by the observation that growth differences were very clear between colonies surrounded by eight neighbors (Figure 2A, crowded) and those surrounded by five neighbors (Figure 2A, uncrowded). Such growth differences became evident after about 2.5 h of incubation (Figure 2B). At 2.5 h, the average colony diameter size was 17 pixels, which is the maximum colony size at which growth differences were not observed. To minimize the neighbor effect, we measured colony mass of the center region within a diameter of 17 pixels (mass*). The growth difference between crowded and uncrowded colonies was dramatically decreased (Figure 2B, mass*), indicating that the neighbor effect is reduced if mass* is used as a growth quantification value.

To evaluate the effect of introducing mass* on colony growth measurements, we assessed both the variation between experiments and reproducibility independent of colony position and plate differences. We calculated the variation among growth measurements for colonies of the same mutants at different positions on the plate. The MGR from 1536 wild-type colonies within a plate exhibited smaller variation in growth values when using mass* (measured using 5 independently prepared plates of wild-type colonies; p < 0.01, t test; Figure 2C), suggesting an improvement of the measurement. For a more realistic evaluation, we examined the variation between multiple growth measurements of shuffled SKO collection plates in which mutants were located at different positions for each measurement. This was done by preparing 4 plates from the Keio collection in which mutants were arrayed at different positions. The intra-class correlation coefficient (ICC) value was used as a variation metric for multiple measurements [18]. The MGR in the case of mass* yielded a higher ICC value (p < 0.01, t test; Figure 2D, shuffle array) than the conventional method (CONV) and MGR-mass, suggesting more reliable measurement. Next, we examined reproducibility as the variation among seven repeats of the growth measurements using standard SKO collection plates (7 independent measurements; see Additional file 3: Table S1). The MGR obtained using mass* exhibited a higher ICC value than when using the conventional method (p < 0.01, t test; Figure 2D, same array), suggesting better reproducibility. Taken together, these results show that the mass* quantification gives improved measurements.

### Comparison of colony-live with the conventional method

**Figure 3:**
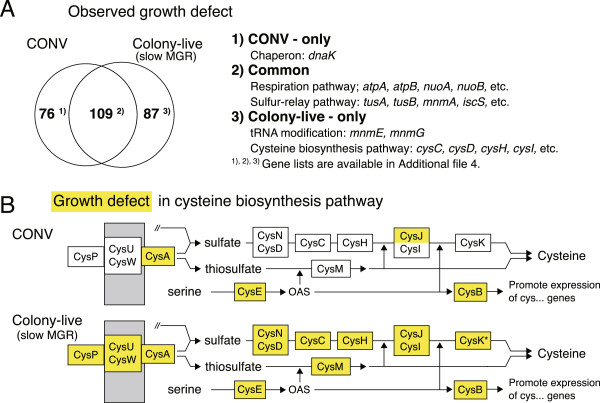
**Comparison of the Colony-live system with the conventional method.** SKO mutants in the Keio collection showing growth defects were statistically identified. Growth values were determined by either the conventional method (CONV) or MGR-mass* value of Colony-live system. **(A)** Observed growth defects of SKO mutants in each subset were confirmed by referring to cited literature or through additional experiments. **(B)** SKO mutants displaying significantly slow growth (yellow) that are disrupted in the cysteine biosynthesis pathway are shown. Genes with an asterisk (such as CysK*) indicate SKO mutants showing normal MGR but low SPG (see Figure 4). Metabolic reaction (solid lines), functional unit (boxes), and inner membrane (grey boxes) are shown. OAS: *O*-acetylserine.

To demonstrate the performance of the Colony-live system, we compared screening results for growth defects in *E. coli* SKO mutants using both the CONV [7] and the Colony-live system. We confirmed that most SKO mutants in the Keio collection did not exhibit severe growth defects, as reported previously for a yeast SKO collection [10]. Statistical selection for growth-defective SKO mutants with a strict threshold (p < 0.01, FDR < 0.01) resulted in a similar number of SKO mutants for both methods: 185 mutants for CONV and 196 for Colony-live (Figure 3A; also see Additional file 4: Document). About 40% of the SKO mutants were commonly selected, and most of these represent deletions of genes that encode proteins involved in the aerobic respiration and sulfur relay pathways of *E. coli*. These SKO mutants are known to show severe growth defects (~1.5- to 2-fold longer doubling time [19-21]), and therefore both measurement methods can identify severe growth-defective SKO mutants in genome-wide screens.

Next, we examined which mutants were differentially selected between the CONV and Colony-live methods. The *mnmE* and *mnmG* mutants were only selected in Colony-live; these SKO mutants are known to show mild growth defects (~1.2-fold longer doubling time) as previously reported [22]. In addition, mutants in all the cysteine biosynthesis pathway genes were selected only by Colony-live (Figure 3B), and the mild growth defects of these mutants were confirmed by further experiments using established accurate growth measurement method with low-density arrayed colonies ([15]; Additional file 1: Figure S8). Therefore, Colony-live can detect mild growth defects that are undetectable by the conventional method. However, the *dnaK* mutant, for example, was selected in CONV but not in Colony-live. Since defective growth of the *dnaK* mutant has been reported previously [23], it appears that Colony-live overlooks some growth-defective mutants. Aberrant colony morphologies have also been observed for membrane-related mutants (*tatA*, *tatB*, *tatC*, *secB*, *rfaE*) and functional unknown mutants (*ycbK*, *yfbN*; see Additional file 5: Table S2). We attempted to quantify the aberrant morphology for DnaK, as an example (see Additional file 1: Figure S2). Because we have not yet developed a general system for quantitating aberrant morphology, we have relied on visual inspection for examining aberrant morphology phenotypes. To improve Colony-live, we believe that quantification of colony morphology is also required and such work is now underway.

### Further classification of SKO mutants by growth kinetics

**Figure 4:**
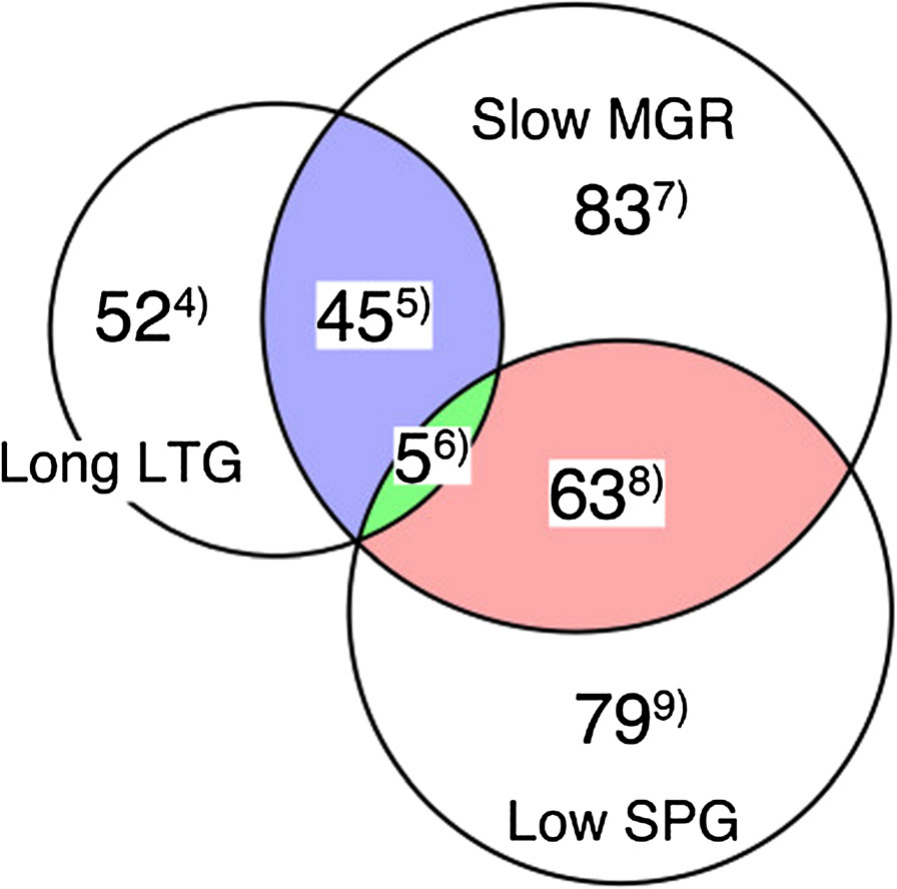
**Classification of MGR-defective mutants by LTG and SPG based on growth kinetics.** SKO mutants showing growth defects in terms of long LTG and low SPG were identified statistically. The intersection of slow MGR and long LTG (blue), slow MGR and low SPG (red), and all of long LTG, slow MGR and low SPG (green) are colored. All of gene lists are listed in Additional file 4.

To demonstrate the utility of the growth kinetics information obtained by Colony-live, we further categorized the slow MGR mutants based on their LTG and SPG values obtained using Colony-live. However, it has been reported that LTG and SPG values depend on its MGR values [24,25], so it was necessary to apply a correction to compare equally among mutants showing different MGR values. To correct these values, the relationships of MGR-LTG and MGR-SPG were estimated by regression analysis using a previously reported theoretical formula ([24,25]; see Additional file 1: Figure S3), and the MGR dependency was subtracted using the regression parameters. After correction, we selected growth defect mutants showing significantly longer LTG or lower SPG by statistical selection with the strict threshold (p < 0.01, FDR < 0.01). Slow MGR mutants were thus classified into two distinct groups: 50 mutants belonged to the long LTG group, and 68 mutants belonged to the low SPG group. Five mutants (*cpxR*, *cydD*, *iscS*, *tolR*, and *rimM* mutants) belonged to both groups (Figure 4; also see Additional file 4: Document). We performed further validation of nine mutants of each of long LTG and low SPG by liquid culture. Two mutants (*rpe, rnt*) in the long LTG group and all nine mutants (*ubiG, ubiH, lpd, atpC, cysE, ubiE, folB, sucA, sucB*) in low SPG group were confirmed. The *tusE* mutant in the long LTG group, which behaved inconsistently in broth cultures, was confirmed by spotting broth suspension on an agar plate (Additional file 1: Figure S8). Differences may result from distinct environmental condition between growth on an agar surface and in liquid medium.

**Figure 5:**
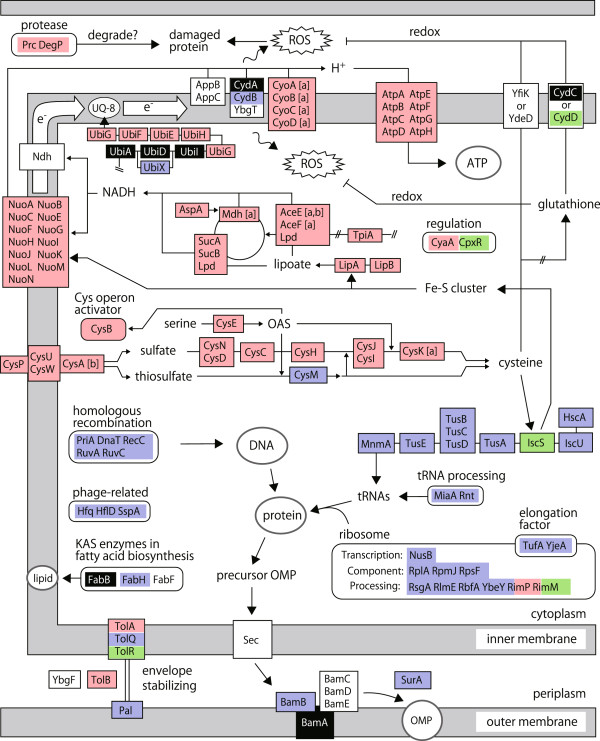
**Global functional map of genes of SKO mutants showing long LTG and low SPG.** Genes screened by the Colony-live method were mapped onto the global map and colored as in Figure 4: long LTG + slow MGR (blue), low SPG + slow MGR (red), or long LTG + low SPG + slow MGR (green) except genes marked [a] with slow SPG with normal MGR, *cysABCD*, *aceEF* and *mdh* and [b] with low SPG with weak significance (p < 0.05, FDR < 0.05). Essential genes in LB (black), reaction direction (solid arrows), functional unit (boxes) and group (rounded boxes), and inner and outer membrane (gray boxes) are shown. ROS: Reactive oxygen species; UQ-8: Ubiquinone-8; OAS: *O*-acetylserine; KAS: β-ketoacyl-ACP synthases; OMP: Outer membrane proteins; Sec: SecYEG translocase.

We also examined gene functions commonly found in the long LTG group or the low SPG group to determine physiologically important functions for early growth and efficient growth, respectively. About 80% of genes in both groups were mapped onto a global functional map based on the knowledge from databases EcoCyc [26] and KEGG [27] (Figure 5). Many genes in the long LTG group were commonly involved in ribosome formation and tRNA modification. On the other hand, most genes in the low SPG group were commonly involved in aerobic respiration (such as NADH dehydrogenase I, cytochrome *bo* terminal oxidase, ATP synthase) and the oxidative stress response system. Thus, both the long LTG group and the low SPG group were linked to different specific physiological functions in *E. coli*.

To illustrate the validity of these results, we evaluated whether mutants impaired for aerobic respiration display lower SPG values. Since the SPG value is the saturation point of cell growth, this value represents the growth yield. Growth yield is known to decrease if genes involved in the aerobic respiration pathway are disrupted. For example, when genes encoding ATP synthase are disrupted, *E. coli* cells resort to less-efficient substrate-level phosphorylation pathways to produce ATP, which reduces growth efficiency [28]. In our screen, almost all genes related to the predominant pathway of aerobic respiration were successfully selected as SPG deficient. Moreover, genes in less-efficient respiration pathways [29], which encode NADH dehydrogenase II (Ndh) and cytochrome *bd*-II terminal oxidase (AppB and AppC), were not selected (Figure 5). This result provides further support for the Colony-live system as a valid tool for acquiring growth kinetics in phenotype classification.

### The importance of cysteine revealed by the colony-live system

The Colony-live system identified new mild growth defects of SKO mutants in the cysteine biosynthesis pathway, which were undetectable by the conventional method. These mild growth defects were unexpected because the availability of cysteine should have been sufficient for two reasons: the experiment was performed on LB agar plates, which supplies most amino acids including cysteine [30], and there are two alternative pathways to synthesize cysteine, which may compensate for each other if either pathway is disrupted (Figure 3B). Most SKO mutants in the cysteine biosynthesis pathway exhibited slow MGR and low SPG in our screen, and the slow MGR and low SPG were confirmed by the further experimentation (data not shown). The slower MGR and lower SPG are a consequence of insufficient supply of cysteine (probably resulting from air oxidation of cysteine to insoluble cystine) because supplementation with substrates related to cysteine synthesis overcame the SPG defect (see Additional file 1: Figure S5). Therefore, sufficient bioavailability of cysteine is required for normal growth of *E. coli*.

## Discussion

In this study, we developed a high-throughput growth measurement system, Colony-live, in order to strike a better balance between throughput and measurement quality. The Colony-live system is designed to provide three growth characteristic values based on growth kinetics (Figure 1), information which is absent in the conventional method. Our system increases the ability to identify unique phenotypes among the wide range of phenotypes in genome-wide studies. Such detailed examination of phenotype alterations allows a deeper understanding of the effects of gene knockout on a cellular system.

To measure growth kinetics accurately and precisely, we focused on reducing the neighbor effect, which can cause reduced measurement quality. We took a new approach to this issue: we referred to the colony growth model as a basis for reducing the neighbor effect. According to the model [31] and experimental observations[32], cells in peripheral regions of a colony replicate faster than cells in the central region. Since the peripheral region becomes closer to neighboring colonies as the colony size increases, the neighbor effect will be more marked in this region as the colony size increases. To minimize the neighbor effect, we derived quantification values of colony mass of the center region (mass*), thereby excluding growth of the peripheral region of large colonies. As expected, the neighbor effect decreased when we took mass* as a quantification value, and the measurement accuracy and precision also successfully improved (Figure 2).The utility of the Colony-live system was tested by genome-wide screening experiments with the Keio collection, in which we found two practical advantages of the Colony-live system over the conventional method. First, owing to the improvement of measurement accuracy and precision, the Colony-live system detected mild growth defect phenotypes, which were undetectable by the conventional method (Figure 3). Under the conditions examined in this study, we found that growth measurement at 20 h incubation time for an average diameter of 17 pixels produced the most accurate growth measurements for Colony-live. For growth under other conditions or other cells, both the incubation time and average diameter for evaluation would need to be determined empirically. Second, owing to the measurement of growth kinetics, the Colony-live system classified two distinct growth alterations, the long LTG group and the low SPG group (Figure 4). Importantly, the functions of knockout genes in each group differed (Figure 5).

The long lag time group (LTG) was linked to two aspects of protein synthesis, the sulfur relay pathway for tRNA modification [21] and ribosome maturation (Figure 5). Why did these mutants display a prolonged lag time? tRNA modification is thought to be functionally important for translational efficiency and fidelity [33], e.g., ribosome maturation. These mutants may prolong the turnaround time of new protein synthesis. The biosynthesis of a large number of new proteins is required during early growth because the global expression profile changes dramatically during the transition from stationary phase to exponential growth phase [34]. Accordingly, the time required for early growth, lag time, was prolonged in these mutants. Since we found other gene classes in the long LTG group, which have functions unrelated to protein synthesis, it is likely that there are multiple mechanisms that can prolong lag time.

The less efficient group (SPG) was linked to aerobic respiration (Figure 5). Interestingly, we found that mutations that disrupt the cysteine biosynthetic pathway also results in a low SPG (see Additional file 1: Figure S5). Our interpretation is that an insufficient supply of cysteine can decrease the activity of the respiration pathway. Physiological connection between cysteine and respiration is that cysteine has defense activity against oxidative stress [35], which is mainly generated by aerobic respiration [36]. In addition, cysteine is required to form Fe-S clusters; essential cofactors of NADH dehydrogenase I, a central enzyme in the respiratory chain. Indeed, ongoing genetic interaction studies within our laboratory using double knockout mutants of *E. coli*, strongly support a functional connection between the cysteine biosynthetic pathway and respiration (unpublished data).

The current Colony-live system has several issues that we need to take into consideration. The first is that morphological abnormalities of the colonies are overlooked. Colony morphology is sometimes changed by SKO mutation, as exemplified by the *dnaK* mutant (see Additional file 1: Figure S2) whose growth defect was missed by the Colony-live system (Figure 3A). Although Colony-live system detected severe and mild growth defects successfully (Figure 3), development of an analysis method to quantify the morphological abnormality of a colony should further enhance the measurement quality. The second issue is the growth effect of light exposure by periodic scanning. All colonies were exposed to strong light for about a second during every scan. Since *E. coli* cells have the potential to sense visible light [37,38] or heat generated, the strong light of the scanner may affect the growth of *E. coli*. We confirmed that the light does not affect growth of individual SKO mutants during scanning (see Additional file 1: Figure S6). However, light and scanner heat have the potential to affect colony growth under specific conditions of interest and should be considered. The optimization of parameters, such as incubation time, diameter of the center region, etc., may be required for growth measurements under other conditions or for other species.

## Conclusions

The Colony-live system provides high-quality growth for genome-wide experiments and enables a deeper understanding of the effects of gene knockouts on cellular systems. Sharing high-quality genome-wide datasets (such as those generated by the Colony-live system) should benefit many researchers who are interested in specific gene functions or the architecture of cellular systems. Our growth data will be publicly available at GenoBase (http://ecoli.naist.jp/GB8). Therefore, the Colony-live system provides a powerful tool to accelerate knowledge accumulation for microbial cells.

## Methods

### Strains and media

All experiments were done using a validated Keio collection for all SKO mutants [16,17] and the wild-type *E. coli* K-12 BW25113 carrying kanamycin-resistant pXX563 (mini-F plasmid, single copy number; unpublished). All SKO mutants were stored in a total of twelve 384-well microtiter plates at −80°C. Cells were grown in Luria-Bertani (LB) medium with 30 μg/ml of kanamycin (Wako, Osaka, Japan). Agar plates were prepared by adding 1.5% agar (Mitsui Sugar, Tokyo, Japan) to LB medium and autoclaving, and 50 ml of the medium was then poured onto a Singer PlusPlate (Singer Instruments, Somerset, United Kingdom). Before use, the agar plate was dried in a laminar flow cabinet for 10–30 min.

### Growth measurement

For the SKO screening experiment, stock plates comprising all the SKO mutants in 384-well-format (24 columns and 16 rows) were thawed at room temperature for about 1 h before use. The liquid cells on the thawed plates were spotted onto fresh LB agar plates with 384-long pins. For the wild-type experiment, *E. coli* K-12 BW25113 was inoculated into 2 ml LB and grown for 20 h at 37°C with shaking. The liquid culture was spotted onto fresh LB agar plates with 384-long pins. After overnight incubation at 37°C, the grown colonies were arrayed from 384-format to 1536-format plates (48 columns and 32 rows) with 384-short pins. These inoculations were performed using a Singer RoToR HDA machine with designated pins (Singer Instruments). After inoculation, colony growth at 37°C was monitored using the Colony-live system. The Colony-live system produced three growth characteristic values (LTG, MGR, and SPG) and also the growth value of the conventional method.

### Colony-live system

The Colony-live system consists of a large incubator (Chromato chamber M-600FN, TAITEC, Osaka, Japan) installed with 12 scanners (GT-X970, Seiko Epson Corp., Nagano, Japan) and two Debian Linux computers (Figure 1A). The scanning program was VueScan version 8.6.66 for Linux and scanning operation was controlled by our automation program. The image and growth analyses described below were performed with our analysis program, which were written in Python 2.7 and integrated to a MySQL5 database. All program codes and detailed information is available at: http://ecoli.naist.jp/Lab/joomla/index.php/en/achievements.

Image analysis of the Colony-live system was performed with OpenCV library [39]. Our image analysis strategy was based on a previous report [15]. The image lighting gradient was corrected according to the reported method [40], and then the image was binarized with Otsu’s thresholding method [41] to determine the positions of arrayed colonies [15]. The colony region was extracted by filtering with the threshold value, which was set to 10-fold greater than standard deviation of background intensities from the mean of background intensities. The colony area was deduced by counting the pixel number within the colony region. Colony mass (*m*) was calculated by integrating the pixel absorbance of all pixels in the colony region:

$$m=\sum_{i=1}^{a}\left(-\log_{10}\left(\frac{I_{i}}{I_{\mathrm{agar}}}\right)\right)$$

where *a* is the total number of pixels in the colony region (equal to the colony area), *I*_*i*_ is the light intensity of the i-th pixel, and *I*_*agar*_ is the mean of light intensities in the surrounding agar region. This formula yields the best quantification performance according to our evaluation (see Additional file 1: Figure S1). Colony mass of the center region (mass*) was calculated by integrating the pixel absorbance of the center region over a diameter of 17 pixels (~1.0 mm) (Figure 1C).

Growth analysis of the Colony-live system was performed with R (http://www.r-project.org) and rpy2 package. After the image analysis, colony growth values less than 1 were set to 1, which is the lower limit quantification value. Colony growth values at all incubation times were normalized by the minimum growth value, and then the first 25 valid growth values exceeding 1 were regressed to the Gompertz growth model to summarize as a three-parameter (*a, b, c*) formula, which is expressed as:

$$G\left(t\right)=a\exp\left(-bc^{t}\right)$$

This regression was performed with the “nls” function in R, and was successful for most growth data unless the colony showed either no growth or extremely poor growth. Using the three parameters, we calculated three growth characters: lag time of growth (LTG), maximum growth rate (MGR), and saturation point of growth (SPG). The formulas for each growth character were derived as reported [9].

$$\mathrm{LTG}=\frac{\ln\left(1/b\right)+1}{\ln\left(c\right)}\;\mathrm{MGR}=-\frac{a\ln\left(c\right)}{e}\;\mathrm{SPG}=a$$

All growth values were normalized using the reported method [7] in the following order: plate-plate normalization, row/column normalization, spatial normalization. Two normalization processes (neighbor effect and batch normalization) were omitted as there was no significant effect on our data.

### Conventional method

The conventional method defines colony growth as colony area after a specified incubation time [6]. In this study the incubation time was fixed at 20 h. The quantities of grown colony areas were determined by the image analysis of the Colony-live system, and these values were normalized using the reported method [7] as described above.

### Correction of LTG and SPG values

To correct LTG and SPG values, the relationships of MGR-LTG and MGR-SPG were estimated. The relationship of MGR-LTG comes from a theoretical study about population lag [24]. When the inoculum amount is high, in accordance with our experimental condition, measured growth lag value (*λ*) depends on growth rate (*μ*) and the mean of individual growth lag (*τ*) as follows:

$$\lambda =\ln\left(1+\mu \tau \right)/\mu$$

The relationship of MGR-SPG comes from studies about maintenance energy [25,42]. Measured growth yield (Y) depends on growth rate (*μ*), maintenance coefficient (*m*), and theoretical growth yield (*Y*_*G*_) as follows:

$$Y=\mu /\left(\frac{1}{Y_{G}}\mu +m\right)$$

Using these formulas, we performed robust regression analysis with the least trimmed squares (LTS) method because many outliers existed in the plot. The LTS method was performed using the “ltgReg” function in the robust base package [43] in R. After the regression, MGR-dependent LTG and SPG values were estimated (see Additional file 1: Figure S3) and were then subtracted from the original values for the correction.

### Statistical analysis

Statistical analysis was performed using R. The intra-class correlation coefficient (ICC) was calculated by one-way ANOVA using the “icc” function in the irr package [44] in R. For the genome-wide data, we used the ranking-based non-parametric test [45] because this method is robust against outliers, which occur in the genome-wide dataset due to human error factors. This test was performed using the “rp” function in the RankProd package [46] in R.

## Abbreviations

LTG: Lag time of growth; MGR: Maximum growth rate; SPG: Saturation point of growth; CONV: Conventional method; SKO: Single-gene knockout; FDR: False-discovery rate; ICC: Intra-class correlation coefficient.

## Competing interests

The authors declare that they have no competing interests.

## Authors’ contributions

RT and HM conceived the study. RT developed the method and performed the experiments. RT, TN, and HM interpreted the data. TT assisted with the discussion on the growth analysis. AM analyzed further evaluation tests. YT and AM prepared download site for programs, raw and processed data, and additional evaluation results. RT, TN, BLW, and HM prepared the figures and wrote the manuscript. All authors read and approved the final manuscript.

## Supplementary Material

Additional file 1: Figure S1 Quantitative performance of a commercial scanner. **Figure S2.** Colony morphology of a *dnaK* mutant. **Figure S3.** Regression analysis for the MGR-LTG and MGR-SPG relationships. **Figure S4.** Correlation between relative nutrient levels and growth yield. **Figure S5.** Restoration of the *cysN* mutant SPG defect by supplementation with 1 mM substrates related to cysteine biosynthesis. **Figure S6.** Effect of light from periodic scanning. **Figure S7.** Validation of growth measurement by liquid culture method. **Figure S8.** Growth measurement with spotting method. **Figure S9.** Growth comparison between the conventional method and Colony-live. Click here for file

Additional file 2 **Movie.** An example of the growth measurement result. Click here for file

Additional file 3: Table S1 Growth values (n = 7) of all *E. coli* SKO mutants. Click here for file

Additional file 4 **Document.** Genes of *E. coli* SKO mutants showing growth defect with statistical significance. Click here for file

Additional file 5: Table S2 Gene list detected only by CONV or Colony-live. Click here for file
