# Supplementary material for: Colony-live —a high-throughput method for measuring microbial colony growth kinetics— reveals diverse growth effects of gene knockouts in Escherichia coli
Source: BMC Microbiol. 2014 Jun 26;14:171. doi: 10.1186/1471-2180-14-171 (PMC4096534; doi:10.1186/1471-2180-14-171)
Supplement: Additional file 1: Figure S1 — Quantitative performance of a commercial scanner. Figure S2. Colony morphology of a dnaK mutant. Figure S3. Regression analysis for the MGR-LTG and MGR-SPG relationships. Figure S4. Correlation between relative nutrient levels and growth yield. Figure S5. Restoration of the cysN mutant SPG defect by supplementation with 1 mM substrates related to cysteine biosynthesis. Figure S6. Effect of light from periodic scanning. Figure S7. Validation of growth measurement by liquid culture method. Figure S8. Growth measurement with spotting method. Figure S9. Growth comparison between the conventional method and Colony-live. [file 1471-2180-14-171-S1.pdf]

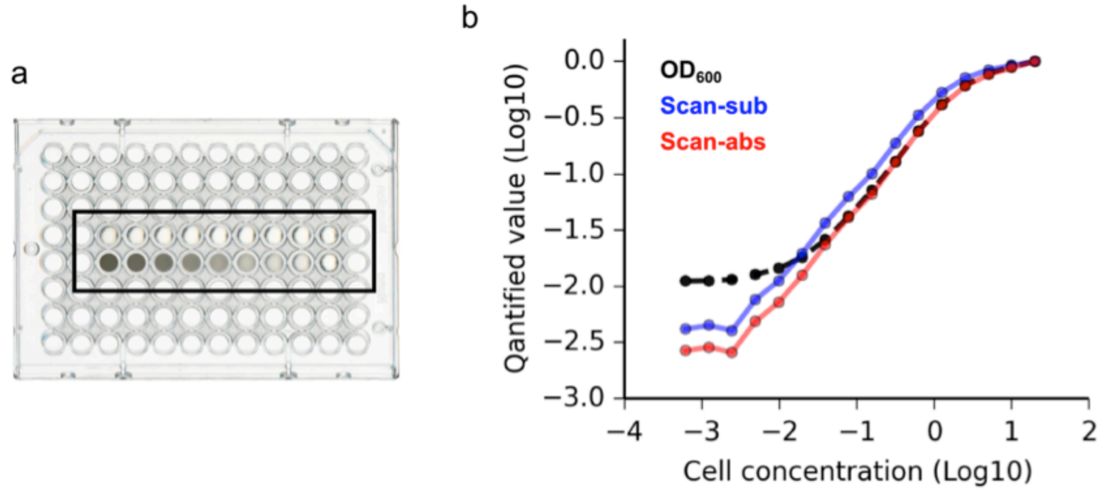

Figure S1: Quantitative performance of a commercial scanner

The quantitative evaluation was performed by optical measurement of series of cell concentration cultures using a commercial scanner (EPSON GT-X970) and a Microplate Reader (SpectraMax Plus384). **(a)** Measurement sample. Overnight *E. coli* culture was concentrated to 20 times by centrifuging and dissolving in LB, and then sequentially 1/2 diluted with LB. Totally 16 different cell concentration samples (20x, 10x, …, 0.0006x) and LB medium were stored in a 96-well microtiter plate. **(b)** Quantification result of the Microplate reader (OD600) and the scanner (Scan-sub and Scan-abs). To compare the result, quantified values were normalized by dividing by the maximum quantified value. For the quantification of scanner, two formulas were used: Scan-sub ; Scan-abs where n is the total number of pixels in the observed region, is the brightness of i-th pixel of the observed region, and is the mean brightness of LB medium.

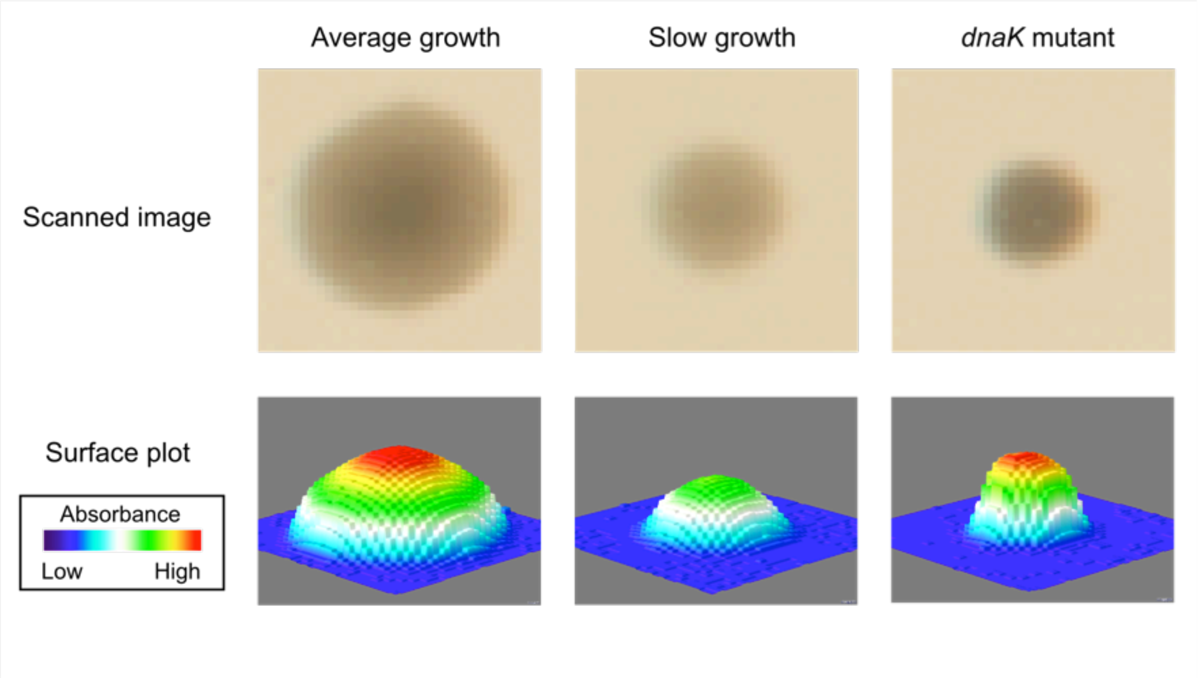

Figure S2: Colony morphology of a *dnaK* mutant

Colony growth of a *gspH* mutant (average growth), an *atpF* mutant (slow growth), and a *dnaK* mutant is shown. (Upper) Grown colony (20-h incubation) images were cropped from the scanned image. (Lower) Surface plots were produced using ImageJ to visualize colony growth in three dimensions. Colors represent pixel absorbance value.

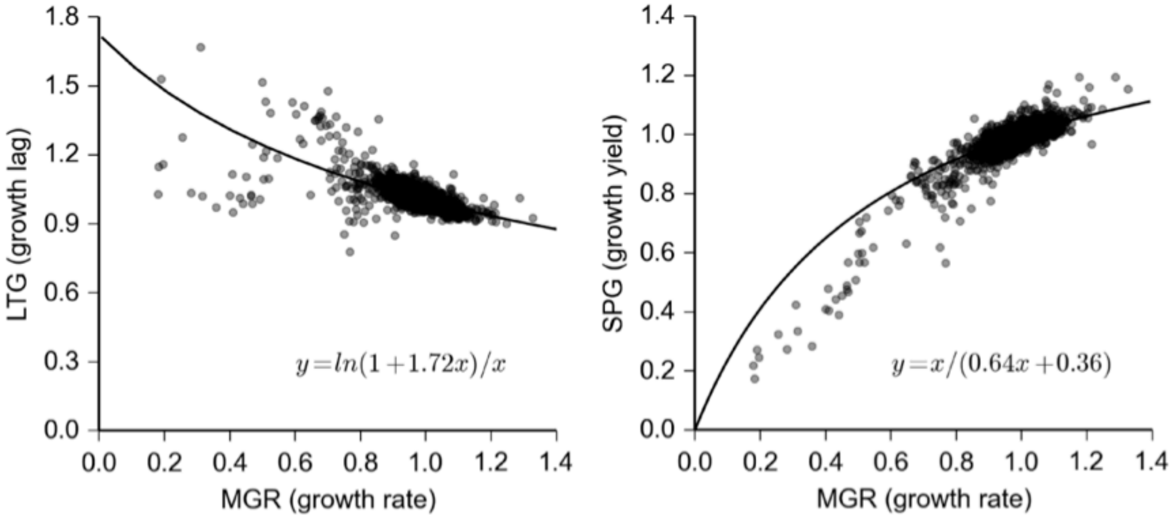

Figure S3: Regression analysis for the MGR-LTG and MGR-SPG relationships.
Each SKO mutant has LTG, MGR, and SPG values from seven individual growth experiments. (Left) Means of MGR and LTG values from all of the SKO mutants are plotted. The regression line and its formula (bottom right in the plot) are shown. (Right) Plot of the mean MGR and SPG values; the format is the same as that in the left plot.

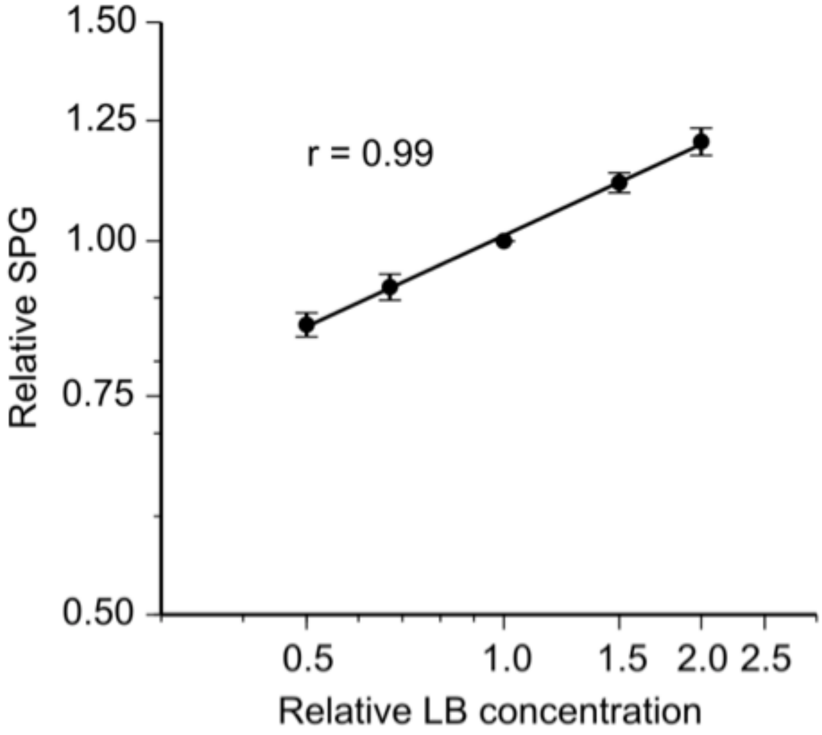

Figure S4: Correlation between relative nutrient levels and growth yield

Wild-type colonies of 1536 array density were grown on agar plates having different LB concentrations (0.5x, 0.67x, 1x, 1.5x and 2x). SPG values at each LB concentration were normalized relative to the SPG values of the identical colony position on standard LB plates (relative LB concentration = 1). Mean and standard deviation (error bars) of relative SPG values of all colonies (n=1536) are shown.

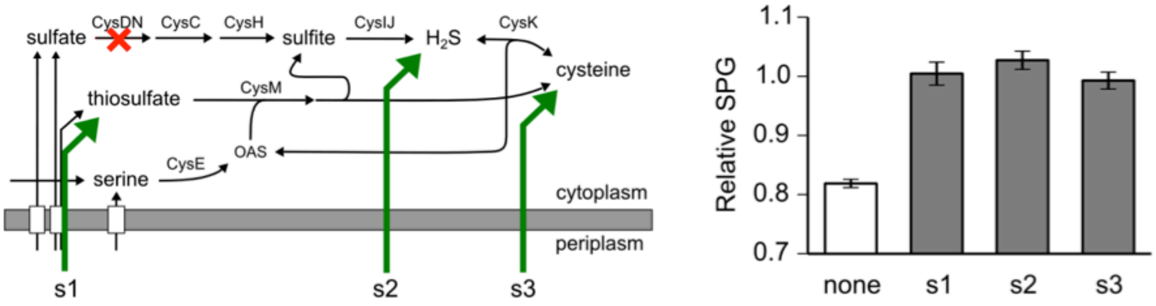

Figure S5: Restoration of the *cysN* mutant SPG defect by supplementation with 1 mM substrates related to cysteine biosynthesis

(Left) The impaired reaction (red cross) and supplemented substrates (green arrows) in the cysteine biosynthesis pathway are shown. s1: thiosulfate; s2: sodium hydrosulfide; s3: cysteine. (Right) Mean and standard deviation (error bars) of relative SPG (*cysN* mutant/ wild-type) were calculated based on multiple measurements (n=10).

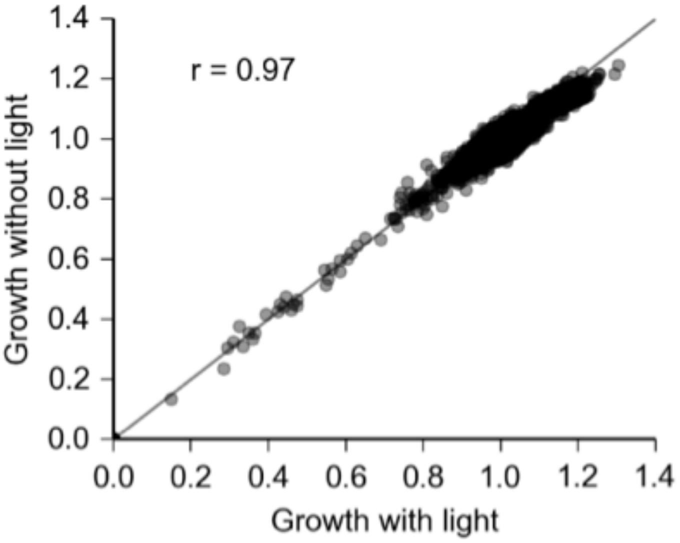

Figure S6: Effect of light from periodic scanning

Colony growth of all the SKO mutants after incubation at 37 ^{O}C for 20 h, with or without scanning during incubation, was compared. Colony growth was quantified by measurement of the mass of the colony center region (mass*).

long LTG

(A)

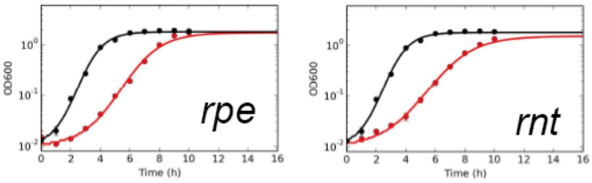

(B)

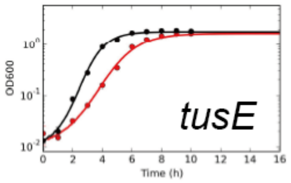

(C)

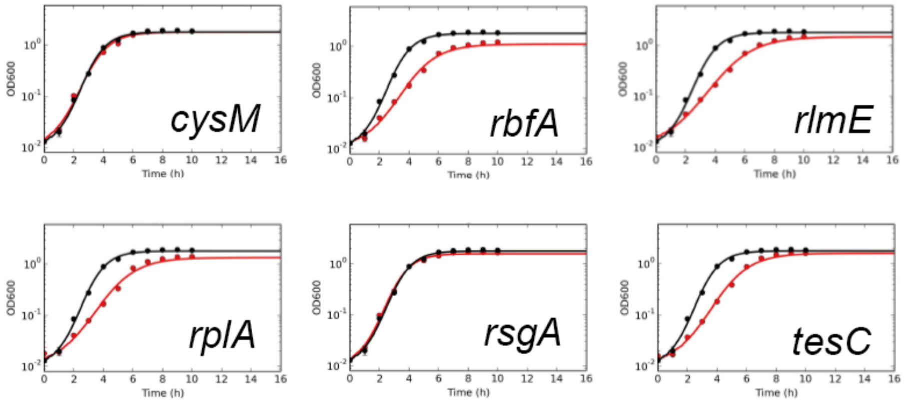

low SPG

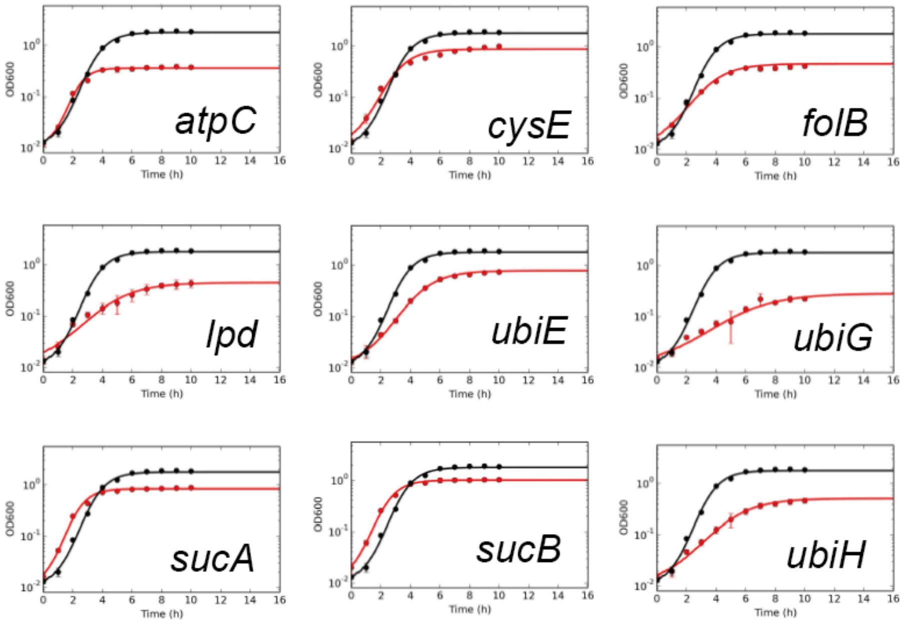

Figure S7: Validation of growth measurement by liquid culture method.
Each of nine candidate deletion mutants of Keio collection in long LTG and low SPG clusters were analyzed their growth profile in LB liquid medium by OD600. Upper panels are shown growth profiles of nine mutants in long LTG group. (A) Confirmed long LTG phenotype in liquid method. (B) Filed to observe long LTG in liquid method, however, using spotting method, fuse showed longer lag-time, shown in Figure S8 b. (C) No longer LTG phenotype. Lower panels show low SPG candidates confirmation by liquid method. All of the candidates were confirmed in liquid method.

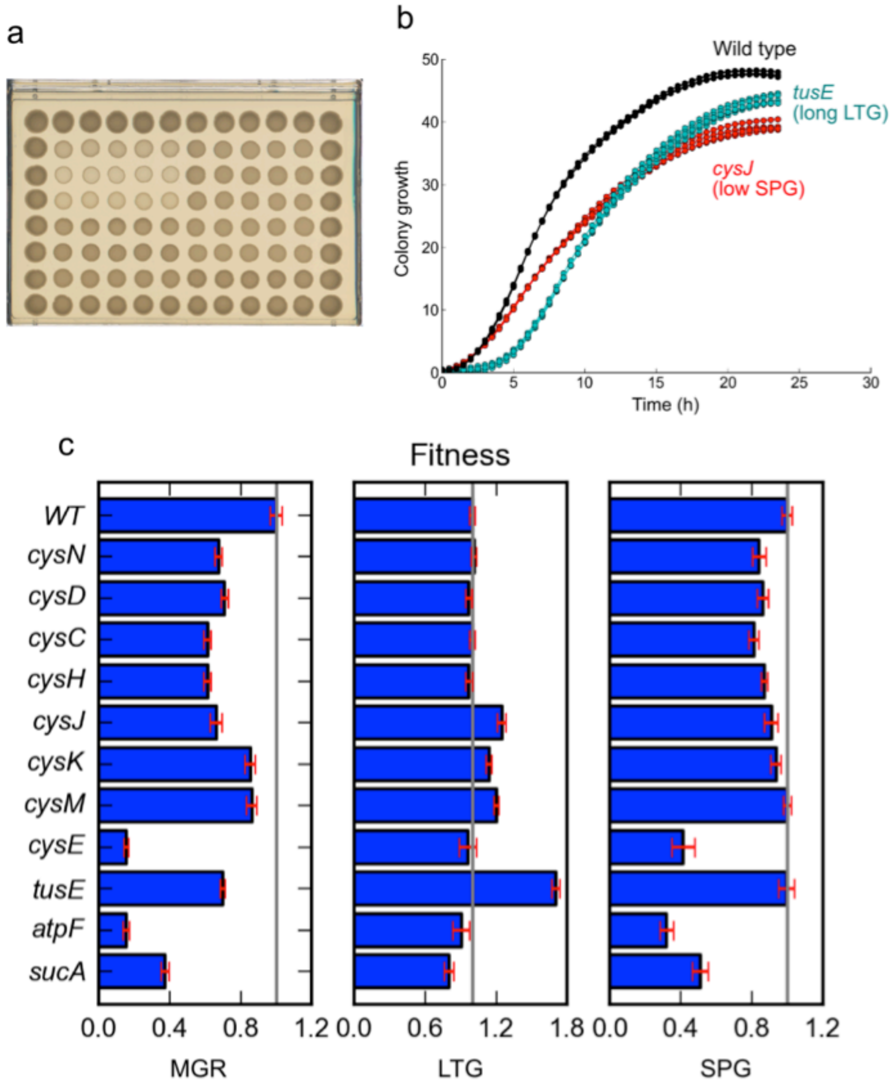

Figure S8: Growth measurement with spotting method.

Growth measurement was performed as previously described (Shah et. al., *BMC Systems Biology* 2007, 1:3). Overnight culture was diluted to OD_{600} = 0.1 in LB, and then spotted 4 uL drops onto LB agar plate. The plates were incubated at 37 ^{O}C for 24 h with periodic scanning. **(a)** Plate image after 24 h incubation. **(b)** Example of growth curves. **(c)** Result of growth analysis. Mean and standard deviation (error bars) of MGR, LTG, and SPG were calculated based on five independent measurements.

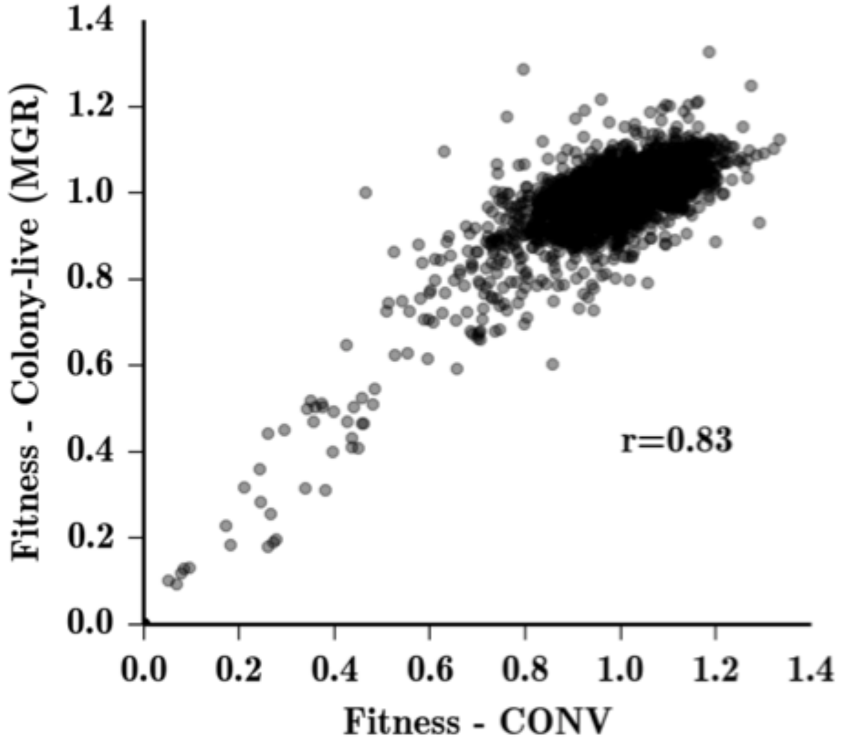

Figure S9: Growth comparison between the conventional method and Colony-live
Colony growth of all SKO mutants were determined by the conventional method (CONV) and Colony-live (MGR). Mean of fitness values were calculated based on seven individual growth experiments.
