# Supplementary material for: Colony-live —a high-throughput method for measuring microbial colony growth kinetics— reveals diverse growth effects of gene knockouts in Escherichia coli
Source: BMC Microbiol. 2014 Jun 26;14:171. doi: 10.1186/1471-2180-14-171 (PMC4096534; doi:10.1186/1471-2180-14-171)
Supplement: Additional file 4 — Document. Genes of E. coli SKO mutants showing growth defect with statistical significance. [file 1471-2180-14-171-S4.docx]

**Mutant list in Fig. 3 and Fig. 4**

Statistical selection result (p<0.01, FDR<0.01).

**== Group 1 ==**

76 mutants in Fig. 3A

*aceE, aceF, acnA, acnB, acpH, appB, argO, aroC, bioA, crr, cvrA, dbpA, dnaK, dsbB, entH, envC, fes, fliI, fryC, ftsP, fumA, fur, glyA, gpmM, hha, holC, htpG, icd, lpcA, mdtH, mpaA, nadA, narW, nudB, pdxB, pdxH, pflA, phoP, pnp, ptsI, quuQ, rarA, rfaD, rfaE, rfaF, rhmR, rppH, secB, sfmA, tatA, tatB, tatC, tig, tpx, yajC, ybhH, ycaC, ycaN, ycbK, ycdY, ycgX, ycjG, ydeP, ydfK, ydhB, ydhI, ydhT, yebG, yfbN, yfdM, yfiM, ygeH, yhbO, yhdU, yjeK, ylaC*

**== Group 2 ==**

109 mutants in Fig. 3A

*aroD, atpA, atpB, atpC, atpD, atpE, atpF, atpG, atpH, bamB, cmk, cpxR, cyaA, cydB, cydD, cyoE, cysA, cysB, cysE, cysJ, dapF, degP, dnaT, envZ, fabH, fkpB, folB, glnA, gmhB, guaA, hflD, hyaD, iscS, iscU, lipA, lipB, lpd, lpxL, manA, mnmA, mtn, nuoA, nuoB, nuoC, nuoE, nuoF, nuoG, nuoH, nuoI, nuoJ, nuoK, nuoL, nuoM, nuoN, nusB, ompR, oxyR, pal, pgm, priA, ratA, rbfA, recA, recB, recC, rimM, rimP, rlmE, rnt, rpe, rplA, rpsF, rsgA, ruvA, ruvC, sdhA, sdhB, smpB, sucA, sucB, sucD, sufA, surA, tolA, tolB, tolQ, tolR, tpiA, trkA, tufA, tusA, tusB, tusC, tusD, tusE, ubiE, ubiF, ubiG, ubiH, ubiX, xerC, xerD, ybeD, ybeY, ybgT, yddE, ydhS, ydjI, yfeS*

**== Group 3 ==**

87 mutants in Fig. 3A

*abgR, allC, aroE, aroK, aspA, betT, carB, ccmA, cysC, cysD, cysG, cysH, cysI, cysM, cysN, cysP, cysU, cysW, ebgR, efp, exuR, fau, fre, ghrA, hfq, hicB, hscA, ihfA, ihfB, intD, kilR, leuE, lpoA, lpp, lrp, marA, miaA, minC, mliC, mnmE, mnmG, mntR, mrcB, mutL, nagA, nlpI, pgl, phr, prc, pyrF, rnhA, rpmJ, rseA, safA, seqA, sppA, srmB, sspA, ssrA, truA, uspE, wbbI, yaaY, ybfB, ybfQ, ybiB, ybjC, ycfH, ycgN, ycgY, ychN, yciH, ycjS, ydhJ, yebE, yecE, yeeN, yeeS, yeiR, yfcR, yfdX, yfiB, ygbM, yggM, yjeA, yliI, zinT*

**== Group 4 ==**

52 mutants in Fig. 4

*ackA, acnA, aroC, cbl, ddpX, dedD, dnaJ, dnaK, dnaQ, efeB, envC, fadR, fdx, gpmM, hipB, lpcA, mdtH, pfkA, pgaC, pgpA, pncA, preT, proW, pta, ptsI, rfaF, rimJ, rimK, rnb, rpoS, rsxC, srlR, tatB, wcaK, yaiY, ybcK, ybgC, ycgE, ycgV, yciB, yciT, ydcS, ydhX, yegS, yfbS, yhcB, yhdU, yjeK, ymfA, ynfA, yohF, ytfK*

**== Group 5 ==**

45 mutants in Fig. 4

*aroD, bamB, carB, cmk, cydB, cysM, dnaT, fabH, glnA, hflD, hfq, hscA, iscU, manA, miaA, mnmA, nusB, pal, priA, rbfA, recC, rlmE, rnt, rpe, rplA, rpmJ, rpsF, rsgA, ruvA, ruvC, sspA, surA, tolQ, tufA, tusA, tusB, tusC, tusD, tusE, ubiX, xerD, ybeD, ybeY, ycgY, yjeA*

**== Group 6 ==**

5 mutants in Fig. 4

*cpxR, cydD, iscS, rimM, tolR*

**== Group 7 ==**

83 mutants in Fig. 4

*abgR, aroE, aroK, betT, ccmA, cyoE, cysA, dapF, ebgR, efp, envZ, exuR, fau, fre, ghrA, hicB, ihfA, ihfB, intD, kilR, leuE, lpp, lpxL, lrp, marA, minC, mliC, mnmE, mnmG, mntR, mrcB, mtn, mutL, nagA, ompR, oxyR, pgl, pgm, phr, pyrF, ratA, recA, recB, rnhA, safA, sdhA, seqA, smpB, sppA, srmB, ssrA, sucD, sufA, trkA, truA,* *uspE, wbbI, xerC, yaaY, ybfB, ybfQ, ybgT, ybiB, ybjC, ycfH, ycgN, ychN, ycjS, yddE, ydhJ, ydhS, yebE, yecE, yeeN, yeiR, yfcR, yfdX, yfeS, yfiB, ygbM, yggM, yliI, zinT*

**== Group 8 ==**

63 mutants in Fig. 4

*allC, aspA, atpA, atpB, atpC, atpD, atpE, atpF, atpG, atpH, cyaA, cysB, cysC, cysD, cysE, cysG, cysH, cysI, cysJ, cysN, cysP, cysU, cysW, degP, fkpB, folB, gmhB, guaA, hyaD, lipA, lipB, lpd, lpoA, nlpI, nuoA, nuoB, nuoC, nuoE, nuoF, nuoG, nuoH, nuoI, nuoJ, nuoK, nuoL, nuoM, nuoN, prc, rimP, rseA, sdhB, sucA, sucB, tolA, tolB, tpiA, ubiE, ubiF, ubiG, ubiH, yciH, ydjI, yes*

**== Group 9 ==**

79 mutants in Fig. 4

*aceF, allA, arcA, aspC, chiP, citG, clpP, cptA, cptB, crcB, cyoA, cyoB, cyoC, cyoD, cysK, dps, eutP, fadM, fbp, flhA, fumA, fur, gcvH, gntR, greA, gstB, guaB, hflC, hns, hyfR, mcbA, mdh, nagK, nhaA, norR, plsY, ptrA, purA, purE, purH, rfaD, rfaG, rfaQ, sdhC, secB, serB, speE, speF, sucC, tesA, tfaR, trpB, xdhD, ybaP, ybcH, ybcN, ybdZ, ybgE, ybhG, yccT, yceJ, ycgL, ydaN, yedF, yeeW, yejG, yejK, yfaE, yfbM, yfcG, yfcP, ygaM, ygcQ, yhbJ, yhbO, yheT, yniB, yrhC, znuB*

**== Further classification of SKO mutants by growth dynamics ==**

# Long LTG: 102 mutants

*ackA, acnA, aroC, aroD, bamB, carB, cbl, cmk, cpxR, cydB, cydD, cysM, ddpX, dedD, dnaJ, dnaK, dnaQ, dnaT, efeB, envC, fabH, fadR, fdx, glnA, gpmM, hflD, hfq, hipB, hscA, iscS, iscU, lpcA, manA, mdtH, miaA, mnmA, nusB, pal, pfkA, pgaC, pgpA, pncA, preT, priA, proW, pta, ptsI, rbfA, recC, rfaF, rimJ, rimK, rimM, rlmE, rnb, rnt, rpe, rplA, rpmJ, rpoS, rpsF, rsgA, rsxC, ruvA, ruvC, srlR, sspA, surA, tatB, tolQ, tolR, tufA, tusA, tusB, tusC, tusD, tusE, ubiX, wcaK, xerD, yaiY, ybcK, ybeD, ybeY, ybgC, ycgE, ycgV, ycgY, yciB, yciT, ydcS, ydhX, yegS, yfbS, yhcB, yhdU, yjeA, yjeK, ymfA, ynfA, yohF, ytfK*

# Slow MGR: 196 mutants

*abgR, allC, aroD, aroE, aroK, aspA, atpA, atpB, atpC, atpD, atpE, atpF, atpG, atpH, bamB, betT, carB, ccmA, cmk, cpxR, cyaA, cydB, cydD, cyoE, cysA, cysB, cysC, cysD, cysE, cysG, cysH, cysI, cysJ, cysM, cysN, cysP, cysU, cysW, dapF, degP, dnaT, ebgR, efp, envZ, exuR, fabH, fau, fkpB, folB, fre, ghrA, glnA, gmhB, guaA, hflD, hfq, hicB, hscA, hyaD, ihfA, ihfB, intD, iscS, iscU, kilR, leuE, lipA, lipB, lpd, lpoA, lpp, lpxL, lrp, manA, marA, miaA, minC, mliC, mnmA, mnmE, mnmG, mntR, mrcB, mtn, mutL, nagA, nlpI, nuoA, nuoB, nuoC, nuoE, nuoF, nuoG, nuoH, nuoI, nuoJ, nuoK, nuoL, nuoM, nuoN, nusB, ompR, oxyR, pal, pgl, pgm, phr, prc, priA, pyrF, ratA, rbfA, recA, recB, recC, rimM, rimP, rlmE, rnhA, rnt, rpe, rplA, rpmJ, rpsF, rseA, rsgA, ruvA, ruvC, safA, sdhA, sdhB, seqA, smpB, sppA, srmB, sspA, ssrA, sucA, sucB, sucD, sufA, surA, tolA, tolB, tolQ, tolR, tpiA, trkA, truA, tufA, tusA, tusB, tusC, tusD, tusE, ubiE, ubiF, ubiG, ubiH, ubiX, uspE, wbbI, xerC, xerD, yaaY, ybeD, ybeY, ybfB, ybfQ, ybgT, ybiB, ybjC, ycfH, ycgN, ycgY, ychN, yciH, ycjS, yddE, ydhJ, ydhS, ydjI, yebE, yecE, yeeN, yeeS, yeiR, yfcR, yfdX, yfeS, yfiB, ygbM, yggM, yjeA, yliI, zinT*

# Low SPG: 147 mutants

*aceF, allA, allC, arcA, aspA, aspC, atpA, atpB, atpC, atpD, atpE, atpF, atpG, atpH, chiP, citG, clpP, cptA, cptB, cpxR, crcB, cyaA, cydD, cyoA, cyoB, cyoC, cyoD, cysB, cysC, cysD, cysE, cysG, cysH, cysI, cysJ, cysK, cysN, cysP, cysU, cysW, degP, dps, eutP, fadM, fbp, fkpB, flhA, folB, fumA, fur, gcvH, gmhB, gntR, greA, gstB, guaA, guaB, hflC, hns, hyaD, hyfR, iscS, lipA, lipB, lpd, lpoA, mcbA, mdh, nagK, nhaA, nlpI, norR, nuoA, nuoB, nuoC, nuoE, nuoF, nuoG, nuoH, nuoI, nuoJ, nuoK, nuoL, nuoM, nuoN, plsY, prc, ptrA, purA, purE, purH, rfaD, rfaG, rfaQ, rimM, rimP, rseA, sdhB, sdhC, secB, serB, speE, speF, sucA, sucB, sucC, tesA, tfaR, tolA, tolB, tolR, tpiA, trpB, ubiE, ubiF, ubiG, ubiH, xdhD, ybaP, ybcH, ybcN, ybdZ, ybgE, ybhG, yccT, yceJ, ycgL, yciH, ydaN, ydjI, yedF, yeeS, yeeW, yejG, yejK, yfaE, yfbM, yfcG, yfcP, ygaM, ygcQ, yhbJ, yhbO, yheT, yniB, yrhC, znuB*

# Slow MGR & Long LTG: 50 mutants

*aroD, bamB, carB, cmk, cpxR, cydB, cydD, cysM, dnaT, fabH, glnA, hflD, hfq, hscA, iscS, iscU, manA, miaA, mnmA, nusB, pal, priA, rbfA, recC, rimM, rlmE, rnt, rpe, rplA, rpmJ, rpsF, rsgA, ruvA, ruvC, sspA, surA, tolQ, tolR, tufA, tusA, tusB, tusC, tusD, tusE, ubiX, xerD, ybeD, ybeY, ycgY, yjeA*

# Slow MGR & Low SPG: 68 mutants

*allC, aspA, atpA, atpB, atpC, atpD, atpE, atpF, atpG, atpH, cpxR, cyaA, cydD, cysB, cysC, cysD, cysE, cysG, cysH, cysI, cysJ, cysN, cysP, cysU, cysW, degP, fkpB, folB, gmhB, guaA, hyaD, iscS, lipA, lipB, lpd, lpoA, nlpI, nuoA, nuoB, nuoC, nuoE, nuoF, nuoG, nuoH, nuoI, nuoJ, nuoK, nuoL, nuoM, nuoN, prc, rimM, rimP, rseA, sdhB, sucA, sucB, tolA, tolB, tolR, tpiA, ubiE, ubiF, ubiG, ubiH, yciH, ydjI, yeeS*

# Slow MGR & Long LTG & Low SPG: 5 mutants

*cpxR, cydD, iscS, rimM, tolR*
